# Economic burden of diabetes mellitus in the WHO African region

**DOI:** 10.1186/1472-698X-9-6

**Published:** 2009-03-31

**Authors:** Joses M Kirigia, Hama B Sambo, Luis G Sambo, Saidou P Barry

**Affiliations:** 1 World Health Organization Regional Office for Africa, Brazzaville, Congo; 2 Manager, Health Financing and Social Protection Programme, World Health Organization, Regional Office for Africa, B.P. 06, Brazzaville, Congo

## Abstract

**Background:**

In 2000, the prevalence of diabetes among the 46 countries of the WHO African Region was estimated at 7.02 million people. Evidence from North America, Europe, Asia, Latin America and the Caribbean indicates that diabetes exerts a heavy health and economic burden on society. Unfortunately, there is a dearth of such evidence in the WHO African Region. The objective of this study was to estimate the economic burden associated with diabetes mellitus in the countries in the African Region.

**Methods:**

Drawing information from various secondary sources, this study used standard cost-of-illness methods to estimate: (a) the direct costs, i.e. those borne by the health systems and the families in directly addressing the problem; and (b) the indirect costs, i.e. the losses in productivity attributable to premature mortality, permanent disability and temporary disability caused by the disease. Prevalence estimates of diabetes for the year 2000 were used to calculate direct and indirect costs of diabetes mellitus. A discount rate of 3% was used to convert future earnings lost into their present values. The economic burden analysis was done for three groups of countries, i.e. 6 countries whose gross national income (GNI) per capita was greater than 8000 international dollars (i.e. in purchasing power parity), 6 countries with Int$2000–7999 and 33 countries with less than Int$2000. GNI for Zimbabwe was missing.

**Results:**

The 7.02 million cases of diabetes recorded by countries of the African Region in 2000 resulted in a total economic loss of Int$25.51 billion (PPP). Approximately 43.65%, 10.03% and 46.32% of that loss was incurred by groups 1, 2 and 3 countries, respectively. This translated into grand total economic loss of Int$11,431.6, Int$4,770.6 and Int$ 2,144.3 per diabetes case per year in the three groups respectively.

**Conclusion:**

In spite of data limitations, the estimates reported here show that diabetes imposes a substantial economic burden on countries of the WHO African Region. That heavy burden underscores the urgent need for increased investments in the prevention and management of diabetes.

## Background

Diabetes mellitus is a disorder caused by insufficient or absent production of the hormone 'insulin' by the pancreas [1]. WHO estimates that more than 180 million people worldwide have diabetes [2]. An estimated 2.9 million people died from diabetes, i.e. a case-fatality rate (CFR) of 0.0161 [3]. In 2000, the prevalence of diabetes in the WHO African Region was estimated at 7.02 million people, out of which about 0.702 million (10%) people had type 1 diabetes and 6.318 million (90%) had type 2 diabetes [2]. About 113,100 people died from diabetes-related causes, 561,600 were permanently disabled, and 6,458,400 experienced temporary disablement.

Diabetes exerts a heavy economic burden on society. This burden is related to health system costs incurred by society in managing the disease, indirect costs resulting from productivity losses due to patient disability and premature mortality, time spent by family members accompanying patients when seeking care, and intangible costs (psychological pain to the family and loved ones).

Barcelo *et al*. [4] estimated the total annual cost associated with diabetes in Latin America and the Caribbean as US$65.216 billion (direct cost US$10.721 billion and indirect cost US$54.495 billion). Shobhana *et al*. [5] estimated that "with a conservative prevalence of 200,000 Type 1 diabetic subjects in India, the cost of treatment could be as high as US$50 million...". The American Diabetes Association [6] estimated that the combined direct and indirect costs of diabetes in 1997 were US$98 billion in the United States of America. Hart, Espinosa and Rovira [7] estimated the total direct costs of diabetes to be over US$650 million in Spain where there were over 1.4 million known diabetics in 1994. Gray and Fenn [8] estimated the cost of type 1 diabetes in England and Wales to be US$1.92 million or US$2042 per person. Unfortunately, there is a dearth of similar evidence for the WHO African Region.

This article focuses on the economic burden of diabetes in the WHO African Region. It attempts to answer the question: from societal perspective (specifically the ministries of health and the families), what is the total cost of diabetes to the Region? The specific objectives were to estimate: (a) the direct costs, i.e. those borne by the health systems and the families in directly addressing the problem; and (b) the indirect costs, i.e. the losses in productivity attributable to premature mortality, permanent disability and temporary disability associated with diabetes.

## Methods

### Overview of health systems in the WHO African region

One of the major threats to economic development confronting the 46 Member States of the WHO African Region is the growing burden of diabetes and other non-communicable diseases. The effectiveness of prevention and control of those diseases hinges largely on the health system performance of its functions of leadership and governance; health workforce; medical products, vaccines and technologies; information; financing; and services delivery.

In the Region, the total number of physicians are 2,281,643; nurses are 3,383,925; midwives are 111,895; dentists are 506,898; pharmacists are 518,378; public and environmental health workers are 177,887; community health workers are 163,285; laboratory technicians are 274,011; other health workers are 1,361,467; and health management and support workers are 2,521,510 [9].

The physician density per 1000 was 0.03–0.78 in 18 countries; 1.01–2.00 in 14 countries; 2.14–3.00 in 4 countries; 3.01–4.00 in 7 countries; and over 4.00 in 3 countries. The nurse density per 1000 was 0.14–0.96 in 17 countries; 1.05–2.00 in 4 countries; 2.10–3.00 in 4 countries; 3.01–4.00 in 8 countries; and over 4.00 in 13 countries. Laboratory technician density varied from minimum of 0.01 per 1000 in Niger to a maximum of 0.65 per 1000 in Cote D'Ivoire [9]. Sixty three percent of the 57 countries experiencing extreme shortages of health workers around the world are in the African Region [10].

About 50% of the population in the Region lack access to essential medicines [10]. The number of hospital beds per 100000 persons among 33 countries that had data was as follows: 8 (24%) countries had 3–20 beds; 13 (39%) countries had 21–40 beds; and 12 (36%) countries had more than 40 beds per 100000 [9].

In 2005, the per capita total expenditure on health in purchasing power parity international dollars (PPP Int$) was $30 and less in 6 countries; $31–60 in 14 countries; $61–90 in 8 countries; $91–120 in 4 countries; and $122–811 in 14 countries. General government expenditure on health as a percentage of total expenditure on health was 30% and less in 7 countries; 31–50% in 13 countries; 51–70% in 17 countries; and 71–90% in 9 countries [11].

The health systems challenges alluded to above have in tandem led to the current situation where 47% of the population in the Region have no access to quality health services [10]. Those health systems bottlenecks also hamper effective response to the growing burden of diabetes and other non-communicable diseases.

### Data

The study used the prevalence estimates of diabetes mellitus for 2000 from a WHO website [2]. The distribution by age from Murray and Lopez [12] was used to disaggregate the total number of people with diabetes into age groups. The gross national income (GNI) per capita in purchasing power parity (PPP) was obtained from a World Bank website [13]. The implied PPP conversion rates used in converting national currencies into current international dollars were from an International Monetary Fund website [14]. The prices of diabetes medicines were obtained from a WHO/AFRO publication [15] and the "hotel" component of hospital costs (i.e. excluding medicines and diagnostic tests) were obtained from a WHO website [16]. The 46 member states in the WHO African Region were classified into three groups using gross national income (GNI) per capita expressed in purchasing power parity for 2005 (Table 1) [13].

**Table 1:** Classification of countries according to gross national income per capita, PPP (at 2005 international dollars) in 2005

Group	GNI per capita (International Dollars, PPP)	Group GNI per capita(International Dollars, PPP)	Countries
1.	≥ 8000	11,113 (standard deviation = 1,950; Median = 10,905)	Botswana, Equatorial Guinea, Gabon, Mauritius, Seychelles, South Africa
2.	2000–7999	3,995 (standard deviation = 1,664; median = 3,865)	Algeria, Angola, Cape Verde, Congo, Namibia, Swaziland
3.	<2000	972 (standard deviation = 437; median = 1020)	Benin, Burkina Faso, Burundi, Cameroon, Central African Republic, Chad, Comoros, Democratic Republic of Congo, Cote d'Ivoire, Eritrea, Ethiopia, Gambia, Ghana, Guinea, Guinea-Bissau, Kenya, Lesotho, Liberia, Madagascar, Malawi, Mali, Mauritania, Mozambique, Niger, Nigeria, Rwanda, Sao Tome and Principe, Senegal, Sierra Leone, Tanzania, Togo, Uganda, Zambia, Zimbabwe

Source: World Bank [10].

* GNI data on Zimbabwe was missing.

Following Barcelo *et al*. [4], the direct costs related to treatment of diabetes patients were obtained from one or more countries in each group from various sources. Each group's average cost figure for various health system inputs and GNI was obtained and used in the analysis. The different countries costs of different inputs used in treatment of diabetes were converted into current international dollar equivalents using PPP conversion rates to ensure that costs for different inputs were similar in each country group. For example, the Mauritian Lipid test cost of Int$28.9 was obtained by dividing the local cost for Lipid profile test R424 by the implied PPP conversion rate of 14.677. A discount rate of 3% was used to convert future indirect cost flows into their present values [4].

### Conceptual framework

**Figure 1:**
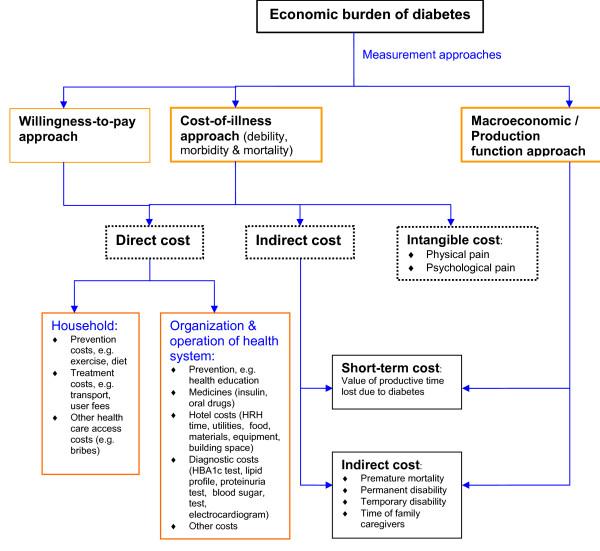
**Conceptual framework of the economic burden of diabetes**.

Figure 1 presents a conceptual framework of the economic burden of diabetes. The figure indicates the three alternative approaches for estimating the economic burden of a public health problem like diabetes. These include: the willingness-to-pay approach [17], the macroeconomic/production function approach [18], and the cost-of-illness [19] approach. This study employed the last approach.

#### Definition of costs estimated

The economic burden of diabetes comprises of direct costs, indirect costs and intangible costs. Direct costs has two components. First, the costs of organizing and operating hospital services: hotel costs (human resources-for-health time, utilities, food, non-pharmaceutical supplies, diagnostic equipment, building space), diagnostic tests (HBA1c test, lipid profile, proteinuria test, blood sugar test, electrocardiogram), medicines (insulin, oral drugs) and devices for injecting insulin (syringes). Second, the out-of-pocket expenses borne by the patients and their families, including health service provider consultation fees, medicines, tests and transport.

The indirect costs consist of opportunity cost of time lost due to morbidity (temporary disability and permanent disability) and premature mortality. The morbidity-related component includes the productivity losses of time invested by patients in outpatient department consultations, travel to and from hospitals, waiting for admission, and during institutionalized treatment; by relatives accompanying patients during pre-admission consultations, travel to and from hospitals accompanying patients, waiting for patients to be admitted, and visiting patients after admission. The premature mortality-related cost component is equal to the lost work-years due to premature death (i.e. retirement age minus age at death) times average remuneration per year.

Intangible costs refer to welfare losses due to the physical and psychological pain. Due to the stigma attached to chronic diseases, the related psychic and social costs to the affected families can be profound. Resource constraints prohibited the collection of willingness-to-pay data that would have facilitated the estimation of intangible costs.

### Analytical model

The total cost (TC) incurred by ministries of health, diabetes patients and family members can be expressed as follows:

*TC *= *DC *+ *IC *+ *ITC*

where: DC is direct cost, IC is indirect cost (which is value of productivity time lost) and ITC is intangible cost (including physical and psychological pain).

### Direct costs

The total direct costs (DC) was estimated using following equations:

*DC *= *TCI *+ *TCS *+ *TCR *+ *TCM *+ *TCD *+ *TCOC *+ *TCH *+ *TCT *+ *OoPE*

where: TCI is total annual cost of insulin; TCS is total annual cost of syringes; TCR is total annual cost of reagent strips; TCM is total annual cost of glucose meters; TCD is total cost of oral drugs; TCOC is total cost of OPD consultations; TCH is total cost of hospitalization; TCT is total cost of diabetes related test for all people with diabetes; and OoPE is the out-of-pocket expenses borne by patients, family members and relatives.

#### Insulin

The total annual cost of insulin for *n*^th ^group of countries (*TCI*_*n*_) was estimated using the following formula: *TCI*_*n *_= (*NII*_*n *_× *AQP *× *P*_*in*_); where: *NII*_*n *_is the total number of patients in need of insulin, which is equal to all the Type 1 diabetes patients plus 5% of the Type 2 diabetes patients; AQP is annual quantity of insulin consumed per patient per year, i.e. 10000 IU [4]; *P*_*in *_is the average price per unit of insulin expressed in international dollars for *n*^th ^group of countries.

#### Syringes

The annual cost of syringes for n^th ^group of countries (*TCS*_*n*_) was obtained as follows:

*TCS*_*n *_= *NII*_*n *_× *DIY *× *P*_*sn*_; where: DIY is the number of days in a year and *P*_*sn *_is the average price per unit of syringe expressed in international dollars for *n*^th ^group of countries.

#### Reagent strips

The annual cost of reagent strips for n^th ^group of countries (*TCR*_*n*_) was obtained as follows:

*TCS*_*n *_= *NII*_*n *_× *TRSU *× *DIY *× *P*_*RSn*_; where: TRSU is the number of times a reagent strip is used in a day, DIY is the number of days in a year and *P*_*RSn *_is the average price per unit of reagent strip expressed in international dollars for *n*^th ^group of countries.

#### Glucose meters

The annual cost of glucose meters for n^th ^group of countries (*TCM*_*n*_) was derived as follows: *TCM*_*n *_= [(*AC*_*n *_× *QGM*_*n*_)/*A*(0.03,5)]; where: *QGM*_*n *_is the quantity of glucose meters needed, i.e. equal to the number of insulin users; *AC*_*n *_is the annual equivalent cost of one glucose meter in international dollars for n^th ^group of countries; and *A*(5,0.03) is the annuity factor calculated assuming a 5 year useful life and a 3% discount rate. The annuity factor was obtained using following formula:  For example, dividing the Group 1 countries average replacement cost of Int$30.97 per glucose meter by the annuity factor yields annual equivalent cost of 30.97/4.579707 = Int$6.762441.

#### Oral drugs

The annual total cost of oral drugs for n^th ^group of countries (*TCD*_*n*_) was calculated as follows: *TCD*_*n *_= *NOD*_*n *_× *NTY *× *P*_*Mn*_; *NOD*_*n *_is the total number of people in need of oral drugs in n^th ^group of countries; *NTY *is the number of 500 mg *Metformin *tablets taken per person per year; and *P*_*Mn *_is the average PPP price per 500 mg *Metformin *tablet in n^th ^group of countries. In our estimation of the TCD, we assumed that 80% of the total population with Type 2 diabetes in each group of countries will need oral drugs and the total number of Metformin tablets needed per person per year would be 1500 [4].

#### Outpatient consultations and hospitalizations

The hospital cost per hospital stay and per outpatient visit represent only the "hotel" component, i.e. excluding drugs and diagnostic tests and including other costs such as personnel, capital and food costs. The "hotel" component of the hospital costs were obtained from a WHO website [16]. The cost of hospital outpatient department consultations (*TCOC*_*n*_) was obtained as follows: *TCOC*_*n *_= *ND*_*n *_× *NV *× *CV*_*n *_; where: *ND*_*n *_is the total number of diabetics in n^th ^group of countries; NV is the total number of OPD visits by a diabetes patient per year in n^th ^group of countries; and *CV*_*n *_is the cost per OPD visit in international dollars in n^th ^group of countries. For example, the TCOC for group 1 countries (NB = 974000, NV = 4, *CV*_*n *_= Int$39.95) was 974000 × 4 × 39.9483333333333 = *Int*$155638707.

The total cost of diabetes patient hospitalizations per year in n^th ^group of countries (*CHOP*_*n*_) was obtained as follows: *CHOP*_*n *_= *NHP*_*n *_× *ALS*_*n *_× *CPID*_*n*_; where: NHP is the total number of patients hospitalized (Type 1 + 5% of Type 2), ALS is the average length of stay and CPID is the cost per inpatient day. We assumed that all people with diabetes will make four visits in a year to a hospital outpatient department [5]. In addition, we assumed that all patients with Type 1 diabetes plus 5% of patients with Type 2 diabetes would require one hospitalization per year [4] and that the average length of stay for diabetes patients is 9 days. For example, the TCOC for group 1 countries (NHP = 146100, ALS = 9.08 CPID = Int$91.10) was 146100 × 9.07979554747842 × 91.1008333333333 = *Int*$120850551.

#### Tests

The total cost of diabetes related tests [4] for n^th ^group of countries (*TCT*_*n*_) was estimated as follows:

*TCT*_*n *_= (*ND*_*n*_) × (*CHBA*_*n *_+ *CLP*_*n *_+ *CECG*_*n *_+ *CPT*_*n *_+ *CBS*_*n*_);

where: *ND*_*n *_is the total number of people with diabetes; *CHBA*_*n *_is the cost of one HBA1c test; *CLP*_*n *_is the cost of one lipid profile; *CECG*_*n *_is the cost of one electrocardiogram; *CPT*_*n *_is the cost of one proteinuria test; and *CBS*_*n *_is the cost of one blood sugar test. We estimated that, in a year, a total of all the 7.02 million people with diabetes would require one HBA_1_c test, one lipid profile test, one *proteinuria *test and one electrocardiogram. The average cost of these tests for group 1 countries was obtained by WHO Country Offices health systems staff from Mauritius; group 2 from Congo, Namibia and Swaziland; and group 3 from Kenya, Mauritania and Senegal. Let us illustrate calculations of TCT using group 1 data: ND = 974000; CHBA = Int$24.77368051; CLP = Int$28.90262726; CECG = Int$16.515787; CPT = Int$8.257893502; and CBS = *Int*$6.193420127. Therefore,

*TCT*_1 _= (974000) × (24.77 + 28.9 +16.52 + 8.26 + 6.19) = *Int*$82,442,680.

#### Household out-of-pocket expenditures

The out-of-pocket expenses borne by patients, family members and relatives in n^th ^group of countries (*OOPE*_*n*_) was obtained using the following formula: *OOPE*_*n *_= (*ND*_*n *_× *APP*_*n*_); where: *ND*_*n *_is the total number of people with diabetes; and *APP*_*n *_is the total annual spending of persons with diabetes on health care provider consultation fees, medicines, tests, transport and other inputs. For example, in group 1 countries (*ND*_1 _= 974000, APP1 = *Int*$45.7406284019997) OOPE was equal to OOPE = 974000 × 45.7406284019997 = *Int*$44,551,372.

The average household out-of-pocket expenditures were obtained from the World Health Survey data [20] on Mauritius for group 1; Congo, Namibia and Swaziland for group 2; and Burkina Faso, Chad, Cote D'Ivoire, Comoros, Ethiopia, Ghana, Kenya, Mali, Mauritania, Malawi, Senegal, Zambia and Zimbabwe for group 3. The household monthly health care expenditures was divided by average household size of 6 members [20] to obtain expenditure per person and then multiplied by 12 to obtain the annual expenditure per person. The result was then divided by the respective country's PPP conversion rate [14] for 2005 to obtain the international dollar equivalent.

### Indirect costs

The total indirect costs (IC) of n^th ^group of countries were obtained using the following algorithm: *IC*_*n *_= (*CTD*_*n *_+ *CPD*_*n *_+ *CPM*_*n *_+ *CPV*_*n*_); where: *CTD*_*n *_is the total cost of productive time lost due to diabetes-related temporary disability; *CPD*_*n *_is the total cost of productive time lost due to permanent disability; *CPM*_*n *_is the total cost of productive time lost due to diabetes-related premature mortality; and *CPV*_*n *_is the productivity loss due to the work time lost by relatives accompanying and visiting patients.

#### Cost of premature diabetes-related mortality (CPM)

A total of 15692, 8636 and 88772 people died from diabetes associated causes in group 1, group 2 and group 3 countries respectively [12]. The distribution of those deaths across the five age brackets was obtained by multiplying the total number of diabetes deaths by the diabetes-related probabilities of death from Murray and Lopez [12]. Those authors provide the average age of onset and the average duration of life lived with diabetes for age brackets 0–4, 5–14, 15–44, 45–59 and 60+ years. The productive life years lost (PLYL) for 15–44, 45–59 and 60+ years age brackets were obtained by subtracting the sum of the average age of onset and average duration of life lived with diabetes from the maximum life expectancy in the African Region. The future PLYL for 0–4 and 5–14 years age brackets were obtained by subtracting the sum of the average age of onset, average duration of life lived with diabetes and 14 years from the maximum life expectancy in the African Region, respectively.

Total cost of premature diabetes-related mortality (CPM) is sum of the cost of premature diabetes-related mortality among persons aged 4 years and less, aged 5–14 years, aged 15–44 years, aged 45–59 years, and aged 60 years and above. The cost of premature diabetes-related mortality among persons of specific age group is the product of number of deaths, total number of productive discounted life years lost (i.e. years above 14 years of age) and gross national income per capita per year (Int$).

In symbolic terms, CPM for i^th ^age bracket can simply be expressed as: ;  is the number of diabetes associated deaths within i^th ^age bracket for n^th ^group of countries; *DPYL*^*i *^is the total number of discounted productive life years lost among persons of i^th ^age bracket; and *GNIPC*_*n *_is the annual gross national income per capita in PPP. The productive life years lost were discounted at a rate of 3% [4].

#### Cost of diabetes-related permanent disability (CPD)

The total cost of productive time lost due to permanent disability (*CPD*_*n*_) is the sum of cost of productive time lost due to permanent disability among persons aged 15–44 years, 45–59 years, and 60 years and over. The non-fatal illness time lost among patients under 4 years and those aged 5–14 years were not costed.

The cost of productive time lost due to permanent disability among persons of various age groups was obtained by multiplying the total number of permanently disabled diabetics (), discounted average duration lived with diabetes (*DAD*) per person, and gross national income per capita per year (*GNIPC*). In symbolic terms, CPD for i^th ^age bracket in ^n^th group of countries can simply be expressed as: .

#### Total cost of productive time lost due to diabetes-related temporary disability (CTD)

Total productive time lost due to diabetes-related temporary disability (CTD) is the sum of cost of productive time lost due to diabetes among persons aged 15–44 years, 45–59 years, and 60 years and over. The non-fatal illness time lost among patients aged 4 years and less and 5–14 years was not costed.

The cost of productive time lost due to diabetes-related temporary disability among persons of various age groups was obtained by multiplying the total number with temporary disability due to diabetes (), days of disability (*DD*) and daily gross national income per capita (*DGNIPC*_*n*_). Symbolically,  for i^th ^age bracket in *n*^th ^group of countries can simply be expressed as: .

#### Cost of productive time lost by caregivers (CPV)

The cost of the work time lost by accompanying/visiting relatives is a product of the number of diabetes cases (ND), number of persons travelling to a health facility (i.e. the accompanying/visiting relatives) (VR), number of days spent visiting a health facility per person per year (NV) and daily gross national income per capita per day (DGNIPC). Symbolically, CPV for i^th ^age bracket in n^th ^group of countries can simply be expressed as: .

The assumptions used in estimating direct and indirect costs can be found in the 'Additional file 1: Data and assumptions used in estimating indirect and direct costs of diabetes in the WHO African Region'. All the cost estimates reported in this paper are in 2005 international dollars, i.e. purchasing power parity.

## Results

### Group 1 countries total costs

Table 2 presents estimates of direct and indirect costs of diabetes in the WHO African Region in 2005 for groups 1, 2 and 3. In group 1 the total number of people with type 1 and type 2 diabetes were estimated at 97400 (10% of total) and 876,600 (90% of total), respectively. The total number of deaths among people with diabetes was estimated at 15,692 (13.9% of total) (see 'additional file 1: Data and assumptions used in estimating indirect and direct costs of diabetes in the WHO African Region').

**Table 2:** Direct and indirect cost of cholera in 2005 international dollars

	GROUP 1		GROUP 2		GROUP 3	
(A). Direct Cost of Diabetes	Sub-Total Cost (Int$)	% of grand total	Sub-Total Cost (Int$)	% of grand total	Sub-Total Cost (Int$)	% of grand total
(1). Total annual cost of insulin	301,619,560	2.71	216,465,240	8.47	3,787,564,806	32.06
(2). Total annual cost of syringes	55,045,570	0.49	21,730,768	0.85	379,961,154	3.22
(3). Total annual cost of reagent strips	79,265,620	0.71	31,292,306	1.22	547,144,062	4.63
(4). Total annual cost of glucose meters	987,900	0.01	390,001	0.02	6,819,144	0.06
(5). Total cost of oral drugs	12,818,831	0.12	12,651,474	0.49	243,016,810	2.06
(6). Total cost of OPD consultations	155,638,707	1.40	78,781,280	3.08	110,057,519	0.93
(7). Total cost of hospitalization	120,850,551	1.09	75,585,203	2.96	128,122,962	1.08
(8). Total cost diabetes related test all people	82,442,680	0.74	48,295,109	1.89	1,036,388,236	8.77
(9). Monetary cost borne by households	44,551,372	0.40	37,992,583	1.49	486,623,596	4.12
						
***Total Direct Cost***	853,220,791		523,183,964		6,725,698,289	
(B) Indirect Cost of Diabetes						
(10). Cost of permanent disability	9,055,480,160	81.33	1,791,388,406	70.06	4,482,451,114	37.94
(11). Cost of temporary disability	109,022,465	0.98	21,567,225	0.84	53,965,981	0.46
(12). Cost of premature deaths among the productive	998,019,608	8.96	197,431,911	7.72	494,018,431	4.18
(13). Productivity loss for care givers	118,623,416	1.07	23,466,521	0.92	58,718,439	0.50
						
**Grand total indirect cost =**	10,281,145,649		2,033,854,063		5,089,153,966	
GRAND TOTAL COST (A+B)	11,134,366,440	100.00	2,557,038,027	100.00	11,814,852,255	100.00

In group 1 the total economic loss attributable to diabetes was Int$11,134,366,440. This estimate consisted of a total direct cost of Int$853,220,791 (7.66%). Out of that direct cost, 35.35% consisted of cost of insulin; 6.45% consisted of cost of syringes; 9.29% consisted of cost of reagent strips; 0.12% consisted of cost of glucose meters; 1.50% consisted of cost of oral drugs; 18.24% consisted of cost of outpatient consultations; 14.16% consisted of cost of hospitalization; 9.66% consisted of cost of diabetes-related diagnostic tests; and 5.22% for health care costs borne by households (patients and their family members) in search of diabetes treatment.

The indirect costs amounted to Int$10,281,145,649 (92.34% of total loss) worth of productive time that was lost by people of the group 1 countries due to diabetes disease. Out of the total indirect cost, about 88.08% was attributed to permanent disability; 1.06% to temporary disability; 9.71% to premature diabetes associated mortality; and 1.15% to productive time lost by family care givers.

### Group 2 countries total costs

In group 2 the total number of people with type 1 and type 2 diabetes were estimated at 53600 and 482400, respectively. The total number of deaths among people with diabetes was estimated at 8636 (7.64% of grand total) (see 'additional file 1: Data and assumptions used in estimating indirect and direct costs of diabetes in the WHO African Region').

The group 2 countries sustained a total economic loss associated with diabetes of Int$2,557,038,027. It consisted of a total direct cost of Int$523,183,964 (20.46%). Out of that direct cost, 41.37% consisted of cost of insulin; 4.15% consisted of cost of syringes; 5.98% consisted of cost of reagent strips; 0.07% consisted of cost of glucose meters; 2.42% consisted of cost of oral drugs; 15.06% consisted of cost of outpatient consultations; 14.45% consisted of cost of hospitalization; 9.23% consisted of cost of diabetes-related diagnostic tests; and 7.26% were health care costs borne by households (patients and their family members) in search of diabetes treatment.

The indirect costs for group 2 countries amounted to Int$2,033,854,063 (79.54% of total loss) worth of productive time. Out of the total indirect cost, about 88.08% was attributed to permanent disability; 1.06% to temporary disability; 9.71% to premature diabetes associated mortality; and 1.15% to productive time lost by family care givers.

### Group 3 countries total costs

In group 3 the total number of people with type 1 and type 2 diabetes were estimated at 551000 and 4959000, respectively. The total number of deaths among people with diabetes was estimated at 88772 (78.5% of grand total) (see 'Additional file 1: Data and assumptions used in estimating indirect and direct costs of diabetes in the WHO African Region').

The group 3 countries sustained a total economic loss associated with diabetes of Int$ 11,814,852,255. It consisted of a total direct cost of Int$6,725,698,289 (56.93%). Out of that direct cost, 56.31% consisted of cost of insulin; 5.65% consisted of cost of syringes; 8.14% consisted of cost of reagent strips; 0.10% consisted of cost of glucose meters; 3.61% consisted of cost of oral drugs; 1.64% consisted of cost of outpatient consultations; 1.90% consisted of cost of hospitalization; 15.41% consisted of cost of diabetes-related diagnostic tests; and 7.24% were health care costs borne by households (patients and their family members) in search of diabetes treatment.

The indirect costs incurred by group 3 countries amounted to Int$5,089,153,966 (43.07% of total loss) worth of productive time. Out of the total indirect cost, about 88.08% was attributed to permanent disability; 1.06% to temporary disability; 9.71% to premature diabetes associated mortality; and 1.15% to productive time lost by family care givers.

### Average costs

Table 3 presents an average cost per case of diabetes for group 1, 2 and 3 countries. The averages in this table were obtained by dividing the itemized total costs in Table 2 by the respective group's number of cases in need of insulin, syringes, reagent strips, glucose meters, oral drugs, hospital outpatient department consultations, hospitalization and diabetes related tests; and numbers of permanently disabled, temporary disabled, premature deaths and care givers.

**Table 3:** Average cost per case of diabetes and diabetes related death (Int$) in 2005

	GROUP 1	GROUP 2	GROUP 3
Cost items	Cost per case diabetes per year	Cost per diabetes case per year	Cost per diabetes case per year
(1). Total annual cost of insulin	2,064.5	2,692.4	4,582.7
(2). Total annual cost of syringes	376.8	270.3	459.7
(3). Total annual cost of reagent strips	542.5	389.2	662.0
(4). Total annual cost of glucose meters	6.8	4.9	8.3
(5). Total cost of oral drugs	15.5	27.8	51.9
(6). Total cost of OPD consultations	159.8	147.0	20.0
(7). Total cost of hospitalization	827.2	940.1	155.0
(8). Total cost diabetes related test all people	84.6	90.1	188.1
(9). Monetary cost borne by households	45.7	70.9	88.3
(10). Cost of permanent disability	116,215.1	41,776.8	10,168.9
(11). Cost of temporary disability	121.7	43.7	10.6
(12). Cost of premature deaths among the productive	63,599.6	22,862.7	5,565.0
(13). Productivity loss for care givers	121.8	43.8	10.7
**GRAND TOTAL COST**	11,431.6	4,770.6	2,144.3

Note: The averages in this table were obtained by dividing the itemized total costs in Table 2 by the respective group's number of cases in need of insulin, syringes, reagent strips, glucose meters, oral drugs, hospital outpatient department consultations, hospitalization and diabetes related tests; and numbers of permanently disabled, temporary disabled, premature deaths and care givers.

The annual cost of insulin in the three groups of countries ranged between Int$2,064.5 – $4,582.7 per diabetes case per year; cost of syringes was Int$270.3 – $459.7 per case; cost of reagent strips was Int$389.2 – $662.0 per case; cost of glucose meters was Int$4.9 – $8.3 per case; cost of oral drugs was Int$15.5 – $51.9 per case; cost of hospital outpatient consultations was Int$20.0 – $159.8 per case; cost of hospital admission was Int$155.0-$827.2 per case; cost diabetes test Int$84.6 – $188.1 per case; health care cost borne by households was US$45.7 – $88.3 per case; cost of productive time lost was Int$10,168.9 – $116,215.1 per permanently disabled case; cost of productive time lost was Int$10.6 – $121.7 per temporarily disabled case; and cost of productive time lost was Int$10.7 – $121.8 per caregiver. Cost of productive time lost per premature death ranged between Int$5,565.0 in group 3 to $63,599.6 in group 1 of countries.

Table 4 shows the average cost per person with diabetes in group 1, 2 and 3. These averages were obtained by dividing the itemized total costs in Tables 2 by 974000, 536000 and 5510000 diabetes cases in group 1, 2 and 3 respectively. The annual cost of insulin was Int$309.7 – $687.4 per diabetes patient; cost of syringes was Int$40.5 – $69.0 per diabetes patient; cost of reagent strips was Int$58.4 – $99.3 per diabetes patient; cost of glucose meters was Int$0.7 – $1.2 per diabetes patient; cost of oral drugs was Int$13.2 – $44.1 per diabetes patient; cost of hospital outpatient consultations was Int$20.0 – $159.8 per diabetes patient; cost of hospital admission was Int$23.3-$141.0 per diabetes patient; cost diabetes test Int$84.6 – $188.1 per diabetes patient; health care cost borne by households was Int$45.7 – $88.3 per diabetes patient; cost of productive time lost was Int$813.5 – $9297.2 per diabetes patient; cost of productive time lost was Int$9.8 – $111.9 per diabetes patient; and cost of productive time lost was Int$89.7 – $1024.7 per diabetes patient. Cost of productive time lost due premature death was Int$2144.3 – $11431.6 per diabetes patient.

**Table 4:** Average cost per person with diabetes (Int$) in 2005

	GROUP 1	GROUP 2	GROUP 3
Summary of direct Cost of Diabetes	Cost per diabetes patient (Int$)	Cost per diabetes patient (Int$)	Cost per diabetes patient (Int$)
(1). Total annual cost of insulin	309.7	403.9	687.4
(2). Total annual cost of syringes	56.5	40.5	69.0
(3). Total annual cost of reagent strips	81.4	58.4	99.3
(4). Total annual cost of glucose meters	1.0	0.7	1.2
(5). Total cost of oral drugs	13.2	23.6	44.1
(6). Total cost of OPD consultations	159.8	147.0	20.0
(7). Total cost of hospitalization	124.1	141.0	23.3
(8). Total cost diabetes related test all people	84.6	90.1	188.1
(9). Monetary cost borne by households	45.7	70.9	88.3
**Total Direct Cost**	876.0	976.1	1,220.6
Summary of Indirect Cost of Diabetes			
(10). Cost of permanent disability	9,297.2	3,342.1	813.5
(11). Cost of temporary disability	111.9	40.2	9.8
(12). Cost of premature deaths among the productive	1,024.7	368.3	89.7
(13). Productivity loss for care givers	121.8	43.8	10.7
Grand total indirect cost =	10,555.6	3,794.5	923.6
**GRAND TOTAL COST**	11,431.6	4,770.6	2,144.3

Note: These averages were obtained by dividing the itemized total costs in Tables 2 by 974000, 536000 and 5510000 diabetes cases in group 1, 2 and 3 respectively.

## Discussion

The 7.02 million cases of diabetes recorded by countries of the African Region in 2000 resulted in a total economic loss of Int$25.51 billion, i.e. $3633 per patient with diabetes. Approximately 43.65%, 10.03% and 46.32% of that loss was incurred by groups 1, 2 and 3 countries, respectively. Group 1 had only six countries that control a relatively substantive amount of wealth, which largely accounts for the high proportion of economic burden of diabetes. Group 3 consisted of 33 countries with the lowest GNI per capita; it bore 78% of the total burden of diabetes in the Region and the highest economic burden of diabetes. Comparatively, the total annual costs associated with diabetes in Latin America and the Caribbean were estimated at US$65.216 billion [4].

The grand total indirect cost was about Int$8.1 billion (32%) in the Region, i.e. 1,154.15 per diabetes patient. The direct cost incurred in treating diabetes was Int$853.2 million in group 1, Int$523.3 million in group 2 and Int$6.7 billion in group 3. The main driver of direct cost across the three groups was the total number of people in need of insulin.

On the other hand, indirect cost of diabetes amounted to Int$10.3 billion in group 1, Int$2.03 billion in group 2 and Int$5.09 billion in group 3. Permanent disability accounted for 81.33%, 70.06% and 37.94% of the total indirect costs in groups 1, 2 and 3 countries, respectively. Intuitively, this is understandable given the chronic nature of diabetes disease. This finding is closely similar to that of Barcelo *et al*. [4] which found that indirect costs accounted for 82% of the total costs in the Latin America and the Caribbean.

The accuracy these estimates hinges on the plausibility of the assumptions contained in the 'additional file 1: Data and assumptions used in estimating indirect and direct costs of diabetes in the WHO African Region'; and their interpretation should be tempered with the limitations highlighted below. The reader should keep in mind that the purpose of total cost of illness studies such as the one reported in this paper is not to guide policy decisions but instead to raise awareness among policy-makers and the public about the negative economic impact of diabetes.

### Limitations of the study

#### a) Disregard of complications in the costing of burden associated with diabetes

Various complications such as retinopathy, cardiovascular diseases, nephropathy and peripheral vascular disease are associated with diabetes [4]. Most of the deaths associated with diabetes results from those complications. Unfortunately, due to lack of information on such complications for the African Region, it was not possible to directly estimate their cost. Thus, it is likely that by disregarding such complications we will have underestimated the economic burden.

#### b) Assumption that those who suffer diabetes disability and mortality would have future earnings

In this study we assumed that all those who are temporarily/permanently disabled by diabetes or die from causes associated with diabetes would have future earnings. This assumption can be contested especially in African countries where the formal sectors are small and hence the proportion of people in formal employment is small. In situations where unemployment rate is high, the marginal productivity of labor might be less than the average. Should this be the case, the use of gross national income per capita might over-estimate the economic burden of diabetes.

#### c) Use of human capital approach

It is an approach that values health benefits in terms of the present value of future lost output (as proxied normally by earnings and other labour costs). This approach values health benefits in terms of production gained due to a decrease in mortality (loss of productive years), morbidity (loss of working time), and debility (loss of productive capacity at work). The use of human capital approach assumes that the objective function that society is trying to maximize through improved health is Gross National Income; and wages in the African Region are a precise indicator of productivity. The approach has been criticized for not being consistent with the basic rationale of the economic calculus used in cost-benefit-analysis, i.e. the potential Pareto optimality; and the fact that people value prevention of premature death, morbidity and debility per se rather than their concern to preserve productive resources and maintain future levels of GNP, among others [21].

#### d) Assumption that all diabetes cases receive diagnostic tests

We have assumed that all diabetes cases receive five diagnostic tests. In reality, not all diabetes patients in the African Region would be able to undergo all the tests. Thus, by assuming that all patients would receive the five tests we may have overestimated the actual cost of diagnosis in the Region.

#### e) Omission of intangible costs

Apart from the physical pain associated with diabetes complications, it has other psychological costs. For example, many communities in Africa may be averse to getting married in families with history of diabetes, and this may have enormous psychological costs on the families concerned. Unfortunately, since majority of the data used in this study were obtained from secondary sources, it was not possible to conduct a household survey that would have made it possible to estimate the intangible costs using contingent willingness-to-pay approach [17,22].

#### f) Use of per capita GNI to value-productive time lost

This study attempted to estimate the loss in the gross national income (GNI) and not the total economic cost of disability and premature mortality associated diabetes. The social value of the contribution that women make to African societies is greater than that captured in GNI calculations. This is because the International Labour Organization's (ILO) definition of labour force includes the employed (including the armed forces), the unemployed, and the first-time job-seekers, but excludes full-time homemakers and other unpaid caregivers and workers in the informal sector. The majority of the women in Africa are either full-time homemakers and/or informal sector workers, and, thus, their invaluable contribution to society is excluded from GNI calculations [23].

## Conclusion

In spite of data limitations, the estimates reported here show that diabetes imposes a substantive economic burden on countries of the African Region. That heavy burden underscores the urgent need for increased investments to fully implement the WHO Regional Committee for Africa [24], World Health Assembly [25,26] and United Nations General Assembly [27] resolutions on the prevention and control of diabetes.

In addition, given the high degree of ignorance about the magnitude of the epidemiological and economic burdens of diabetes in the WHO African Region, there is an urgent need for further research to determine:

• national-level epidemiological burden of diabetes, measured in terms of its prevalence, incidence, mortality, and, probably, disability-adjusted life years lost;

• national-level economic burden of diabetes, broken down by different productive and social sectors and occupations of patients and relatives; and

• national-level costs and effectiveness of alternative preventive, diagnostic and treatment interventions of diabetes to aid choice of cost-effective strategies.

## Competing interests

The authors declare that they have no competing interests.

## Authors' contributions

JK, LS, BS and SB participated equally in the design, analysis and writing of the manuscript. All the authors read and approved the final manuscript.

## Pre-publication history

The pre-publication history for this paper can be accessed here:

http://www.biomedcentral.com/1472-698X/9/6/prepub

## Supplementary Material

Additional File 1 Appendix: Data and assumptions used in estimating indirect and direct costs of diabetes in the WHO African Region. Click here for file
