# Supplementary material for: Economic burden of diabetes mellitus in the WHO African region
Source: BMC Int Health Hum Rights. 2009 Mar 31;9:6. doi: 10.1186/1472-698X-9-6 (PMC2674592; doi:10.1186/1472-698X-9-6)
Supplement: Additional File 1 — Appendix: Data and assumptions used in estimating indirect and direct costs of diabetes in the WHO African Region. [file 1472-698X-9-6-S1.doc]

**Appendix: Data and assumptions used in estimating indirect and direct costs of diabetes in the WHO African Region**

| **Item** | **Group 1** | **Group 2** | **Group 3** | **Source of data** |
| --- | --- | --- | --- | --- |
| **A). Population** |
| (1).Total No. of people with diabetes mellitus | 974,000 | 536,000 | 5,510,000 | WHO [2] |
| (2). No. of people with Type 1 diabetes mellitus (10% of total) | 97,400 | 53,600 | 551,000 |
| (3). No. of people with Type 2 diabetes mellitus (90% of total) | 876,600 | 482,400 | 4,959,000 |
| **B). Mortality**  (1). No. of deaths related to diabetes (Total No. of people with diabetes mellitus X 0.0161) | 15,692 | 8,636 | 88,772 | Murray and Lopez [9] |
| (2).No. of deaths in 0-4 (8%) | 1,255 | 691 | 7,102 |
| (3). No. of deaths in 5-14 (8%) | 1,255 | 691 | 7,102 |
| (4). No. of deaths in 15-44 (8%) | 1,255 | 691 | 7,102 |
| (5) No. of deaths in 45-59 (16%) | 2,511 | 1,382 | 14,204 |
| (6). No. of deaths in 60+ (60%) | 9,415 | 5,181 | 53,263 |
| (7). No. of discounted future PLYL: 0-4 per person | 26.77 | 26.77 | 26.77 | Murray and Lopez [9] |
| (8). No. of discounted future PLYL: 5-14 per person | 24.52 | 24.52 | 24.52 |
| (9). No. of discounted future PLYL: 15-44 per person | 10.63 | 10.63 | 10.63 |
| (10). No. of discounted future PLYL: 45-59 per person | 4.58 | 4.58 | 4.58 |
| (11). No. of discounted future PLYL: 60+ per person | - | - | - |
| (12). Average gross national income per capita in international dollars (PPP) | 11,113 | 3,995 | 972 | World Bank [10] |
| ***(13). Cost (Int$, PPP)*** | ***991,772,539*** | ***196,196,093*** | ***490,926,140*** |  |
|  |  |  |  |  |
| **C). Permant Disability**  (1). No. permanently disabled by diabetes (8%) = | 77,920 | 42,880 | 440,800 | WHO [2]   Murray and Lopez [9] |
| (2). No. of 0-4 permanently disabled | 40 | 22 | 224 |
| (3). No. of 5-14 permanently disabled | 40 | 22 | 224 |
| (4). No. of 15-44 permanently disabled | 12,035 | 6,623 | 68,083 |
| (5). No. of 45-59 permanently disabled | 26,687 | 14,686 | 150,971 |
| (6). No. of 60+ permanently disabled | 39,119 | 21,527 | 221,297 |
| (7). Discounted years of permanent disability: 0-4 years per person | 0.61 | 0.61 | 0.61 | Murray and Lopez [9] |
| (8). Discounted years of permanent disability: 5-14 years per person | 2.83 | 2.83 | 2.83 |
| (9). Discounted years of permanent disability: 15-44 years per person | 19.60 | 19.60 | 19.60 |
| (10). Discounted years of permanent disability: 45-59 years per person | 12.56 | 12.56 | 12.56 |
| (11). Discounted years of permanent disability: 60+ years per person | 6.23 | 6.23 | 6.23 |
| (12). Average gross national income per capita in international dollars (PPP) | 11,113 | 3,995 | 972 | World Bank [10] |
| ***(13). Cost (Int$, PPP)*** | ***9,055,480,160*** | ***1,791,388,406*** | ***4,482,451,114*** |  |
|  |  |  |  |  |
| **D). Temporary Disability**  (1). No. with temporary disabled by diabetes = | 896,080 | 493,120 | 5,069,200 | WHO [2]   Murray and Lopez [9] |
| (2). No. of 0-4 temporarily disabled patients | 456 | 251 | 2,580 |
| (3). No. of 5-14 temporarily disabled patients | 456 | 251 | 2,580 |
| (4). No. of 15-44 temporarily disabled patients | 138,402 | 76,164 | 782,953 |
| (5). No. of 45-59 temporarily disabled patients | 306,902 | 168,890 | 1,736,169 |
| (6). No. of 60+ temporarily disabled patients | 449,864 | 247,564 | 2,544,919 |
| (7). Days of temporary disablement per person per year | 4 | 4 | 4 | Personal communication with Regional Advisor for Diabetes |
| (8). Daily GNI per capita (PPP) | 30.45 | 10.95 | 2.66 | World Bank [10] |
| ***(9). Cost (Int$, PPP)*** | ***109,022,465*** | ***21,567,225*** | ***53,965,981*** |  |
| **E). Productivity loss among care givers**  (1). No. of diabetics patients | 974,000 | 536,000 | 5,510,000 | WHO [2] |
| (2). No. accompanying patients to health facilities | 1 | 1 | 1 | Personal communication with Regional Advisor for Diabetes |
| (3).No. of health facility visits per year | 4 | 4 | 4 | Personal communication with Regional Advisor for Diabetes |
| (4). Daily GNI per capita (PPP) | 30.45 | 10.95 | 2.66 | World Bank [10] |
| ***(5). Cost (Int$, PPP)*** | ***118,623,415.53*** | ***23,466,521*** | ***58,718,439*** |  |
| **DIRECT COSTS OF DIABETES** |  |  |  |  |
| **(F). Insulin**  (1). Number of people with Type 1 diabetes (10% of Regional total) | 97,400 | 53,600 | 551,000 | WHO [2] |
| (2). 5% of people with Type 2 diabetes need insulin | 48,700 | 26,800 | 275,500 | Barcelo et al [4] |
| (3). Total number in need of insulin | 146,100 | 80,400 | 826,500 |  |
| (4). Annual use of 10000 IU for each patient taking insulin | 10,000 | 10,000 | 10,000 | Barcelo et al [4] |
| (5). Total quantity of insulin needed per year | 1,461,000,000 | 804,000,000 | 8,265,000,000 |  |
| (6). Group average cost of insulin (per IU) in PPP | 0.206 | 0.269 | 0.458 | WHO/AFRO [12] |
| ***Total annual cost of insulin*** | ***301,619,560.163*** | ***216,465,240*** | ***3,787,564,806*** |  |
|  |  |  |  |  |
| **(G). Syringes**  (1). Total number in need of insulin | 146,100 | 80,400 | 826,500 | Barcelo et al [4] |
| (2). No. of times a syringe is needed per day | 3 | 3 | 3 |  |
| (3). No. of days in a year | 365 | 365 | 365 |  |
| (4). Cost of one syringe | 1.032 | 0.741 | 1.260 | NIH [20] |
| ***Total annual cost of syringes*** | ***165,136,709.19*** | ***65,192,304*** | ***1,139,883,462.83*** |  |
|  |  |  |  |  |
| **(H).Reagent strips**  (1). No. of patients in need of reagent strips | 146,100 | 80,400 | 826,500 |  |
| (2). No. of times a strip is used per day | 3 | 3 | 3 | Personal communication with Regional Advisor for Diabetes |
| (3). No. of days in a year | 365 | 365 | 365 |  |
| (2). Cost of one reagent strip | 0.495 | 0.355 | 0.605 | NIH [20] |
| ***Total annual cost of reagent strips*** | ***79,265,620*** | ***31,292,306*** | ***547,144,062*** |  |
|  |  |  |  |  |
| **(I). Glucose meters**  (1). Quantity of glucose meters needed | 146,100 | 80,400 | 826,500 |  |
| (2). Useful life (years) | 5 | 5 | 5 | NIH [20] |
| (3). Replacement price (US$) | 31 | 22 | 38 |  |
| (4). Annuity factor | 5 | 5 | 5 | NIH [20] |
| (5). Annual cost of one glucose meter | 6.8 | 4.9 | 8.3 | Internet. |
| ***Total annual cost of glucose meters*** | ***987,900*** | ***390,001*** | ***6,819,144*** |  |
|  |  |  |  |  |
| **(J). Oral drugs**  (1). 80% of total population with Type 2 diabetes used oral drugs | 827,900 | 455,600 | 4,683,500 | WHO [2]  Barcelo et al [4] |
| (2). Number of tablets taken per person per year | 1,500 | 1,500 | 1,500 | Barcelo et al [4] |
| (3). Total number of tablets needed per year | 1,241,850,000 | 683,400,000 | 7,025,250,000 |  |
| (4).Cost per tablet | 0.0103224 | 0.0185125 | 0.0345919 | WHO/AFRO [12] |
| ***Total cost of oral drugs [3 x 4]*** | ***12,818,831*** | ***12,651,474*** | ***243,016,810*** |  |
|  |  |  |  |  |
| **(K). Cost of OPD Consultations**  (1). Total number of diabetics | 974,000 | 536,000 | 5,510,000 | WHO [2] |
| (2). Number of OPD visits per patient per year | 4 | 4 | 4 | Personal communication with Regional Advisor for Diabetes |
| (3).Total number of OPD visits | 3,896,000 | 2,144,000 | 22,040,000 |  |
| (4). Cost per outpatient visit | 39.95 | 36.75 | 4.99 | WHO [13] |
| ***Total OPD consultation cost*** | ***155,638,707*** | ***78,781,280*** | ***110,057,519*** |  |
|  |  |  |  |  |
| **(L). Cost of Hospitalizations**  (1). Total number of patients hospitalized (100% of type 1 + 5% of type 2 diabetes) | 146,100 | 80,400 | 826,500 | WHO [2]  Barcelo et al [4] |
| (2). Average length of hospital stay for diabetes patients | 9 | 9 | 9 | Shobhana et al [5] |
| (3). Total number of inpatient days | 1,326,558 | 730,016 | 7,504,451 |  |
| (4). Unit cost per bed day at levels 1, 2 & 3 hospitals | 91.10 | 103.54 | 17.07 | WHO [13] |
| ***Total cost of hospitalization*** | ***120,850,551*** | ***75,585,203*** | ***128,122,962*** |  |
|  |  |  |  |  |
| **(M). Cost of diabetes tests**  (1). Total number of people with diabetes | 974,000 | 536,000 | 5,510,000 |  |
| (2). One HBA test | 25 | 28 | 55 | WHO Country Offices health systems advisors |
| (3).One lipid profile | 29 | 30 | 66 |
| (4). One electrocardiogram | 17 | 15 | 30 |
| (5). One proteinuria test | 8 | 10 | 21 |
| (6). Blood sugar | 6 | 7 | 16 |
| ***Total cost diabetes related test all people]*** | 82,442,680 | **48,295,109** | ***1,036,388,236*** |  |
|  |  |  |  |  |
| **(N).Monetary cost borne by households**  (1). Number of diabetics | 974,000 | 536,000 | 5,510,000 | WHO [2] |
| (2). Annual spending on health per person | 46 | 71 | 88 | WHO [17] |
| ***Total cost borne by households annually*** | ***44,551,372.06*** | ***37,992,583.15*** | ***486,623,596*** |  |
